# Supplementary material for: Development of an Enzyme-Linked Immunosorbent Assay (ELISA) for the Quantification of ARID1A in Tissue Lysates
Source: Cancers (Basel). 2023 Aug 14;15(16):4096. doi: 10.3390/cancers15164096 (PMC10452747; doi:10.3390/cancers15164096)
Supplement: Supplementary file 1 [file cancers-15-04096-s001.zip › Supplemental Table S1.pdf]

Supplemental Table S1. Comparison of different commercial ELISA Kits for ARID1A with our ELISA. 11 Columns show the different features of 11 commercial ELISA Kits, that are online available. In order to compare them to our ELISA, we calculated our price by summing up the costs for each component and listed several validation parameters. Still there are some cells where information was not available (NA). Date of creation: 7.20.2023

Company	Catalog no.	Method	Samples	Price/ 96 wells	Sensitivity	Range	Precision – Intra-assay	Precision – Inter-assay	References	Link
Hinsberger et al. 2023		Indirect	Cell culture extracts, tissue homogenates, recombinant standards	188.6 €[1] or 188.6 € + 16.5 €[2] per Lysate	0.39 ng/ml	0.78 – 50 ng/ml	4.1 % 7 samples (n=4)	Standards: 4.5% 8 samples (n=2) on 11 plates; 'same lysate': 10.6% 18 samples (n=2)	-	-
Abcam	ab302760	Sandwich	Cell culture extracts	670 €	0.041 ng/ml	1.71 – 110 ng/ml	7.4% 1 sample (n=8)	8% 1 sample (n=3)	None	https://www.abcam.com/products/elisa/arid1a-elisa-kit-ab302760.html
Abbexa	abx358515	Sandwich	Tissue homogenates, cell lysates and other biological fluids	587.50 €	0.47 ng/ml	0.78 – 50 ng/ml	<10% 3 samples (n=20)	<10% 3 samples (n=8) on 3 plates	None	https://www.abbexa.com/human-arid1a-elisa-kit-1
MyBioSource[3]	MBS2540332	Sandwich	Serum, plasma and other biological fluids	540 US $	0.47 ng/mL	0.78 – 50 ng/mL	3 samples (n=20)	3 samples (n=8) on 3 plates	None	https://www.mybiosource.com/human-elisa-kits/arid1a-at-rich-interactive-domain-containing-protein-1a/2540332
LSBio	LS-F65695	Sandwich	Tissue homogenates, serum, plasma and other biological fluids	980 US $	To be determined	To be determined	NA	NA	None	https://www.lsbio.com/elisakits/mouse-arid1a-baf250-custom-elisa-kit-ls-f65695/65695?trid=247
BT Lab	E3933Hu	Sandwich	Serum, plasma, cell culture supernates	458 US $	6.61 ng/L	12.5 – 800 ng/L	5% 3 samples, (n=18)	NA	None	https://www.bt-laboratory.com/index.php/Shop/Index/productShijiheDetail/p_id/11212.html
BT Lab	E6528Hu	Sandwich	Serum, plasma, cell culture supernates	458 US $	7.89 ng/L	15 – 3000 ng/L	<6% 3 samples, (n=18)	NA	None	https://www.bt-laboratory.com/index.php/Shop/Index/productShijiheDetail/p_id/11880.html
Biorbyt[4]	orb1209952	Sandwich	Serum, plasma, cell	655.40 €	6.61 ng/L	12.5 – 800 ng/L	NA	NA	None	https://www.biorbyt.com/human-at-rich-interactive-

			culture supernates							domain-containing-protein-1a-elisa-kit-orb1209952.html
Biorbyt[4]	orb1209286	Sandwich	Serum, plasma, cell culture supernates	655.40 €	7.89 ng/L	15 – 3000 ng/L	NA	NA	None	https://www.biorbyt.com/human-at-rich-interactive-domain-containing-protein-1a-elisa-kit-orb1209286.html
Krishgen	KBH3933	NA	Serum, plasma, cell culture supernatant	~300 € (France)	Under revalidation	Under revalidation	NA	NA	None	https://www.krishgen.com/product/details/Human-AT-rich-interactive-domain-containing-Protein-1A-ARID1A-ELISA
Krishgen	KBH6528	Sandwich	Serum, plasma, cell culture supernatant	~300 € (France)	7.89 ng/L	15 ng/l – 3000 ng/l	CV<8%	CV<10%	None	https://www.krishgen.com/product/details/KBH6528-Human-AT-rich-interactive-domain-containing-Protein-1A-ARID1A-ELISA

[1]Costs for our ELISA: Plate (750-0083, VWR, Germany): 100 € (60x) -> 1x = 2 €; Fragment (ABN-H00008289-Q01, Abnova, Taiwan): 520 € (25µg, 0.2 µg/µl - 125µl) - 1:4000 respectively 0.5µl -> 2 €; Prim. Ab (ab182560, Abcam, UK): 580 € (100 µl) -> 10 µl = 58 €; Sec. Ab (K4003, Dako, Denmark): 2369 € (110 ml) -> 5ml = 107.7 €; Casein (34006, Thermo Fisher Scientific, USA): 110 € (30g) -> 0.1g = 0.4 €; OPD: 200 € (50x) -> 1x = 4 €; Stable Peroxide (34062, Thermo Fisher Scientific, USA): 83.25 € (100ml) -> 1 ml = 8.3 €; H_2SO_4 (258105, Sigma-Aldrich, USA): 40 € (100 ml) -> 1.5 ml = 0.6 €; Washing solution (VWR, A9177.0100): 372 € (100x, 100*200 ml) -> 300 ml = 5.6 €;

[2] Costs for each Lysate: Fractionation (87790, Thermo Fisher Scientific, USA): 608 € (50x) -> 1x = 12.16 €; Precellys (10144-496, VWR, Germany): 217 € (50x) -> 1x = 4.34 €;

[3] Presumably the same ELISA than abbexa; [4] Presumably the same ELISA than BT Lab
