# Supplementary figures and images for: Development of an Enzyme-Linked Immunosorbent Assay (ELISA) for the Quantification of ARID1A in Tissue Lysates
Source: Cancers (Basel). 2023 Aug 14;15(16):4096. doi: 10.3390/cancers15164096 (PMC10452747; doi:10.3390/cancers15164096)

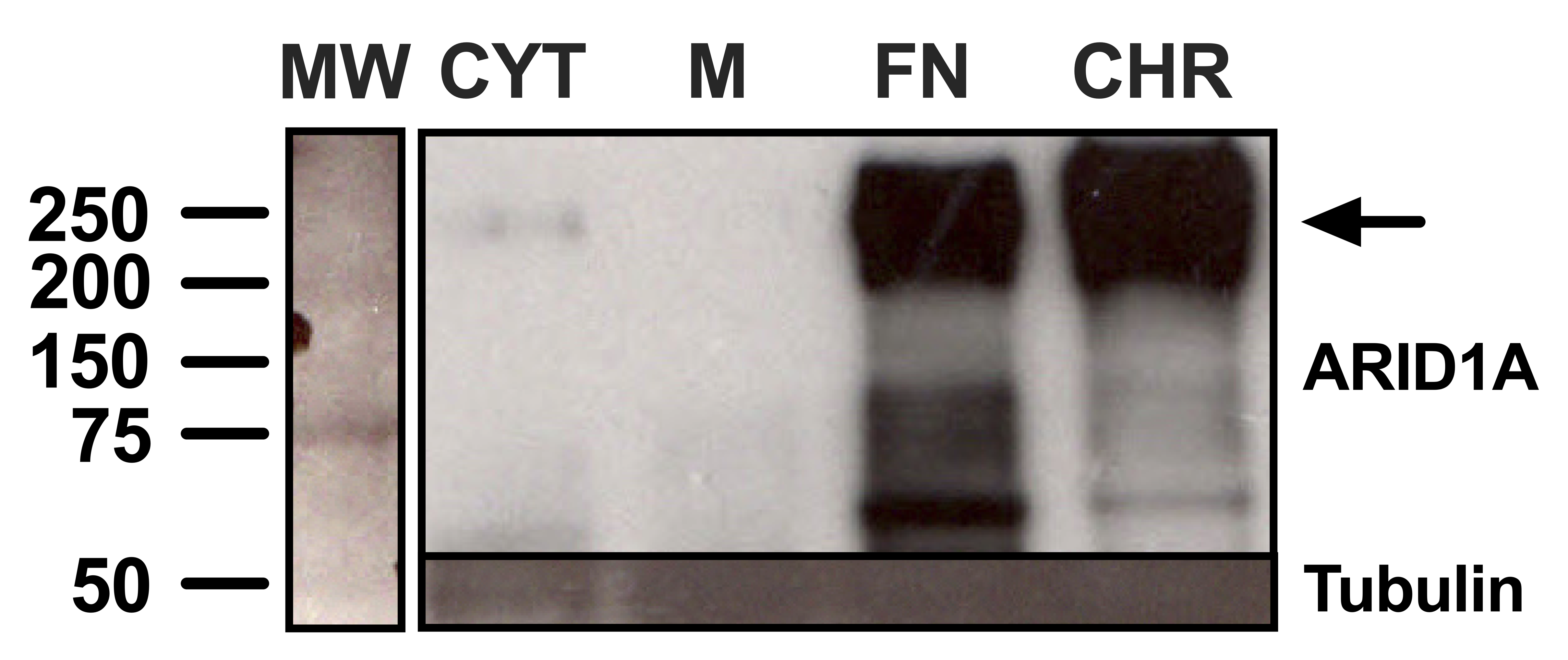

Supplement: Supplementary file 1 [file cancers-15-04096-s001.zip › Figure S1.tiff]

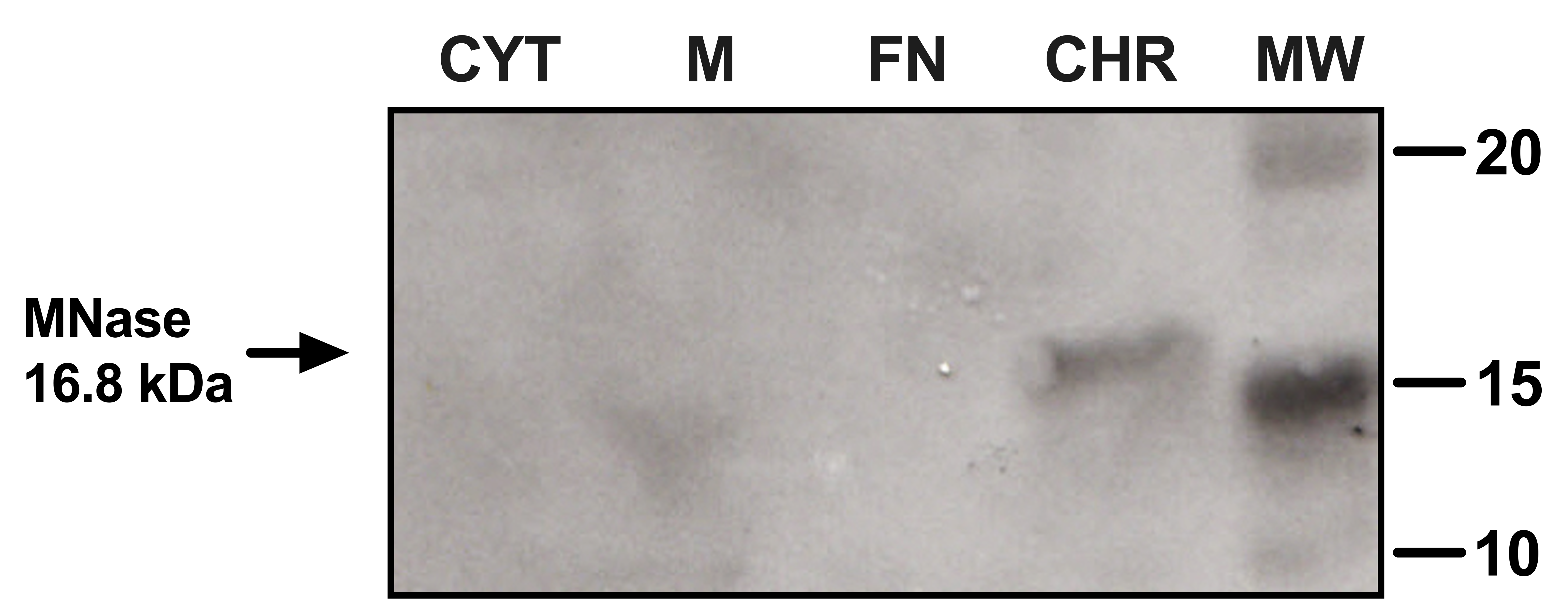

Supplement: Supplementary file 1 [file cancers-15-04096-s001.zip › Figure S2.tiff]

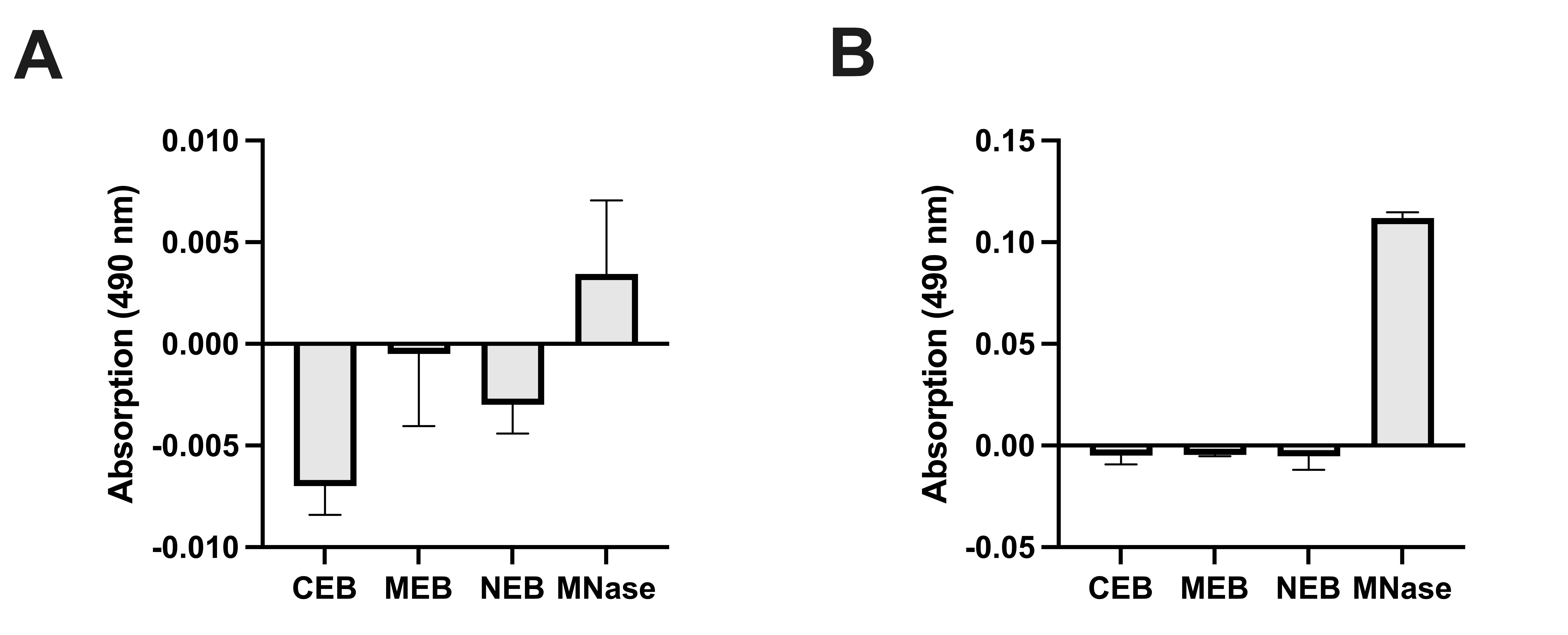

Supplement: Supplementary file 1 [file cancers-15-04096-s001.zip › Figure S3.tiff]

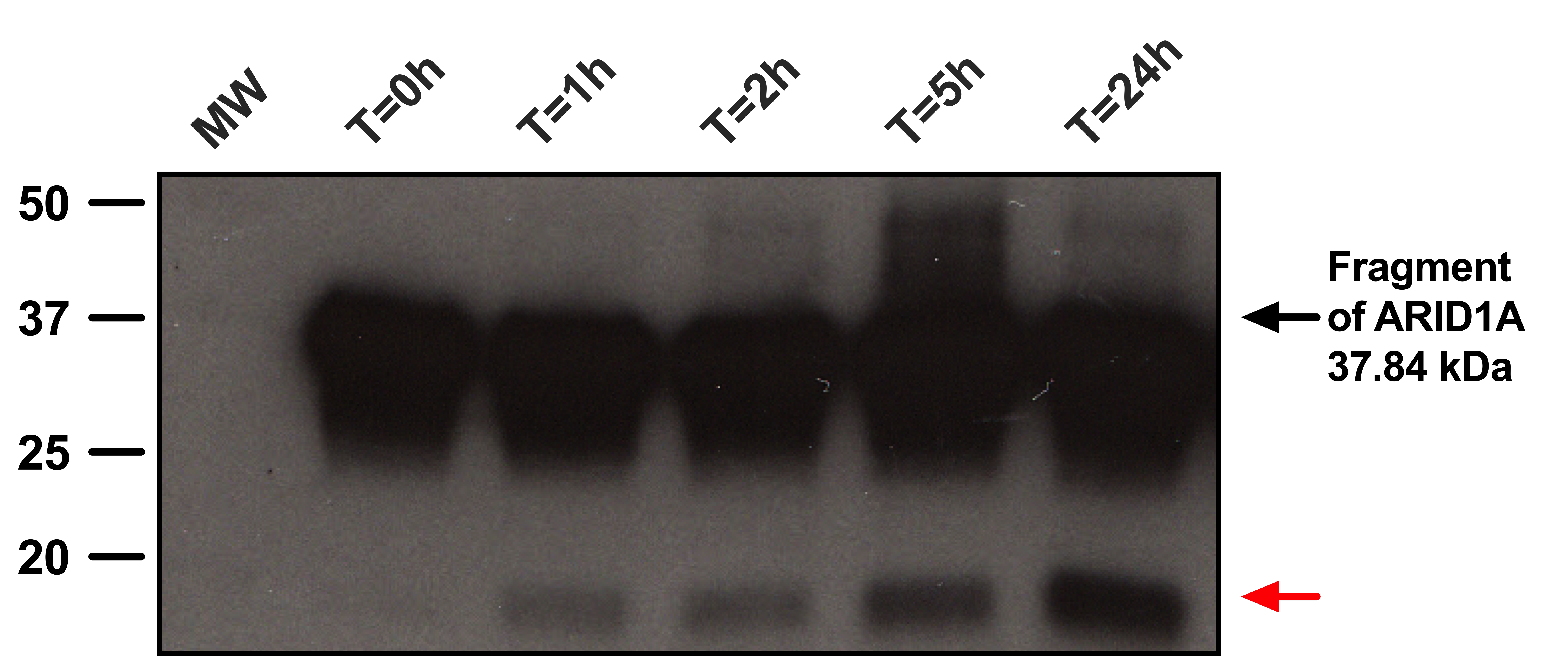

Supplement: Supplementary file 1 [file cancers-15-04096-s001.zip › Figure S4.tiff]

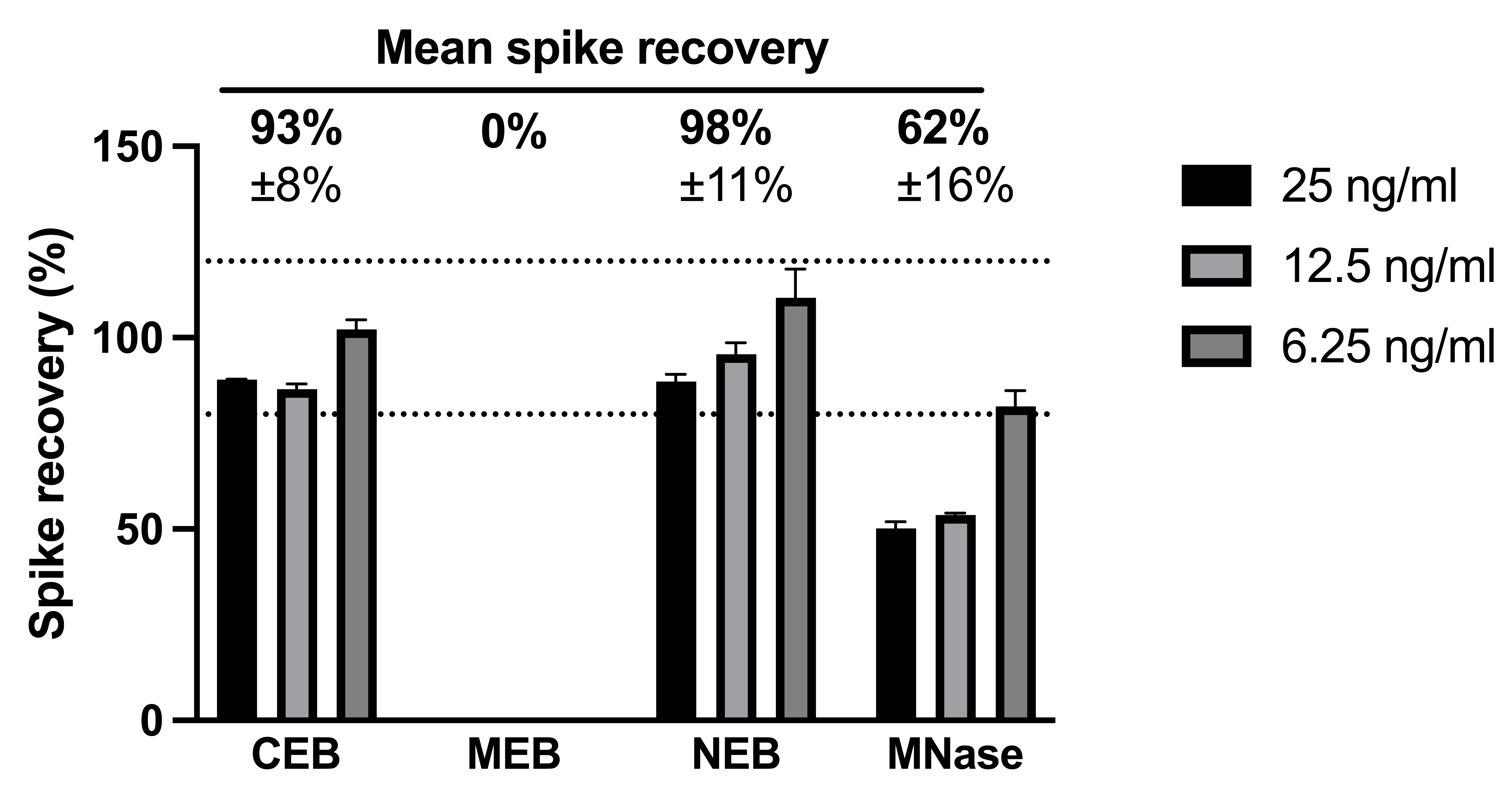

Supplement: Supplementary file 1 [file cancers-15-04096-s001.zip › Figure S5.tiff]

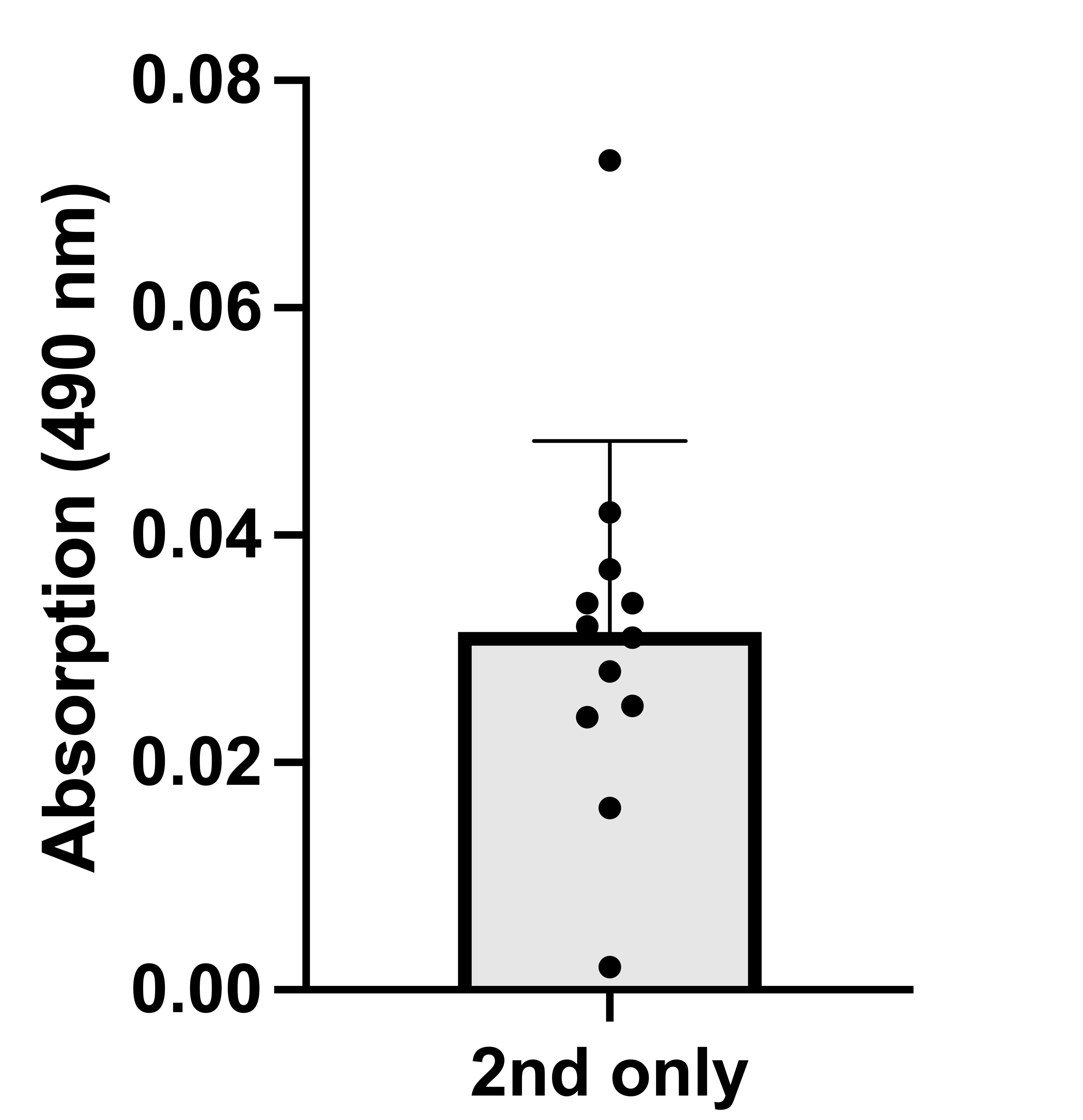

Supplement: Supplementary file 1 [file cancers-15-04096-s001.zip › Figure S6.tiff]

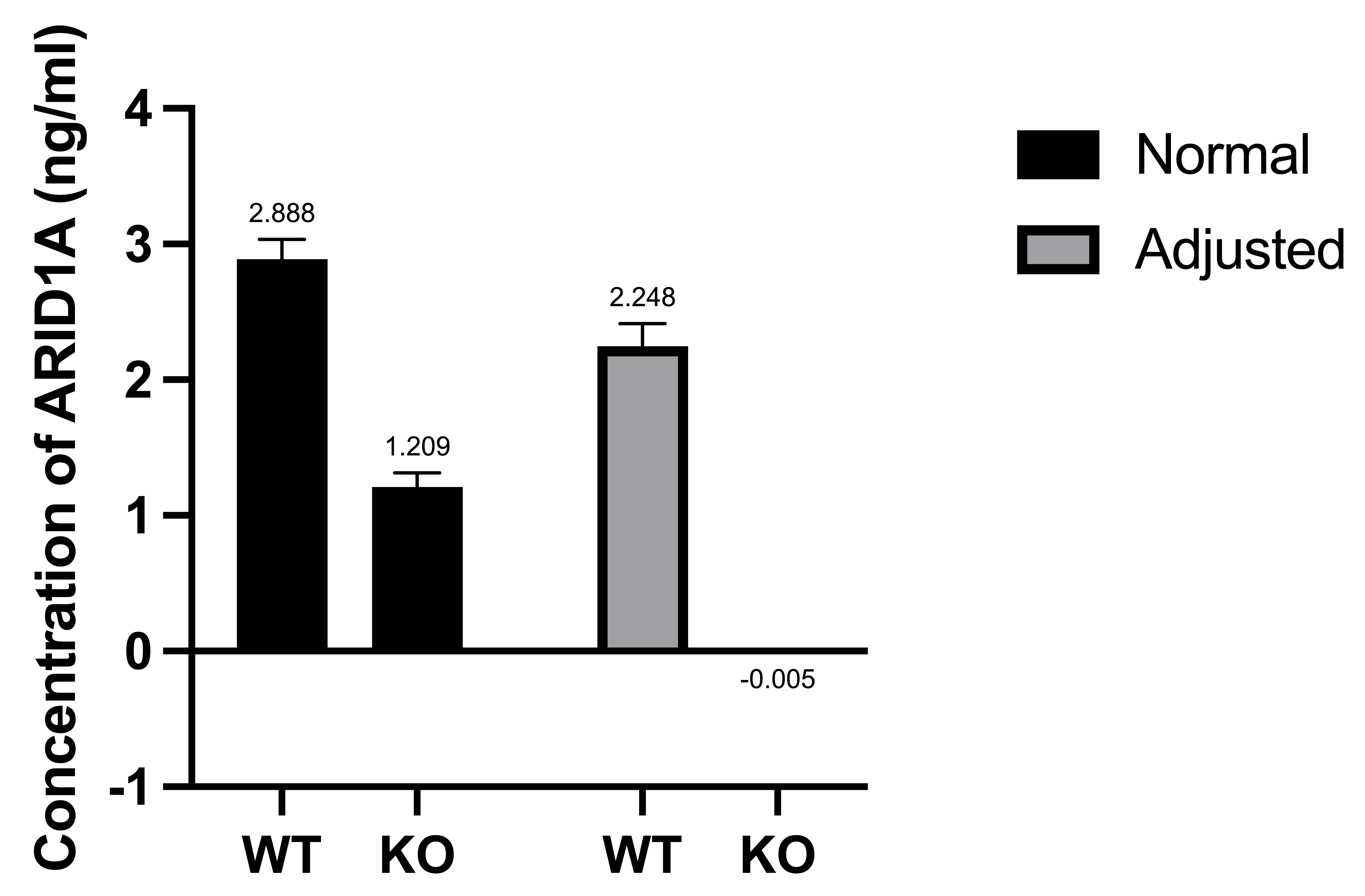

Supplement: Supplementary file 1 [file cancers-15-04096-s001.zip › Figure S7.tiff]

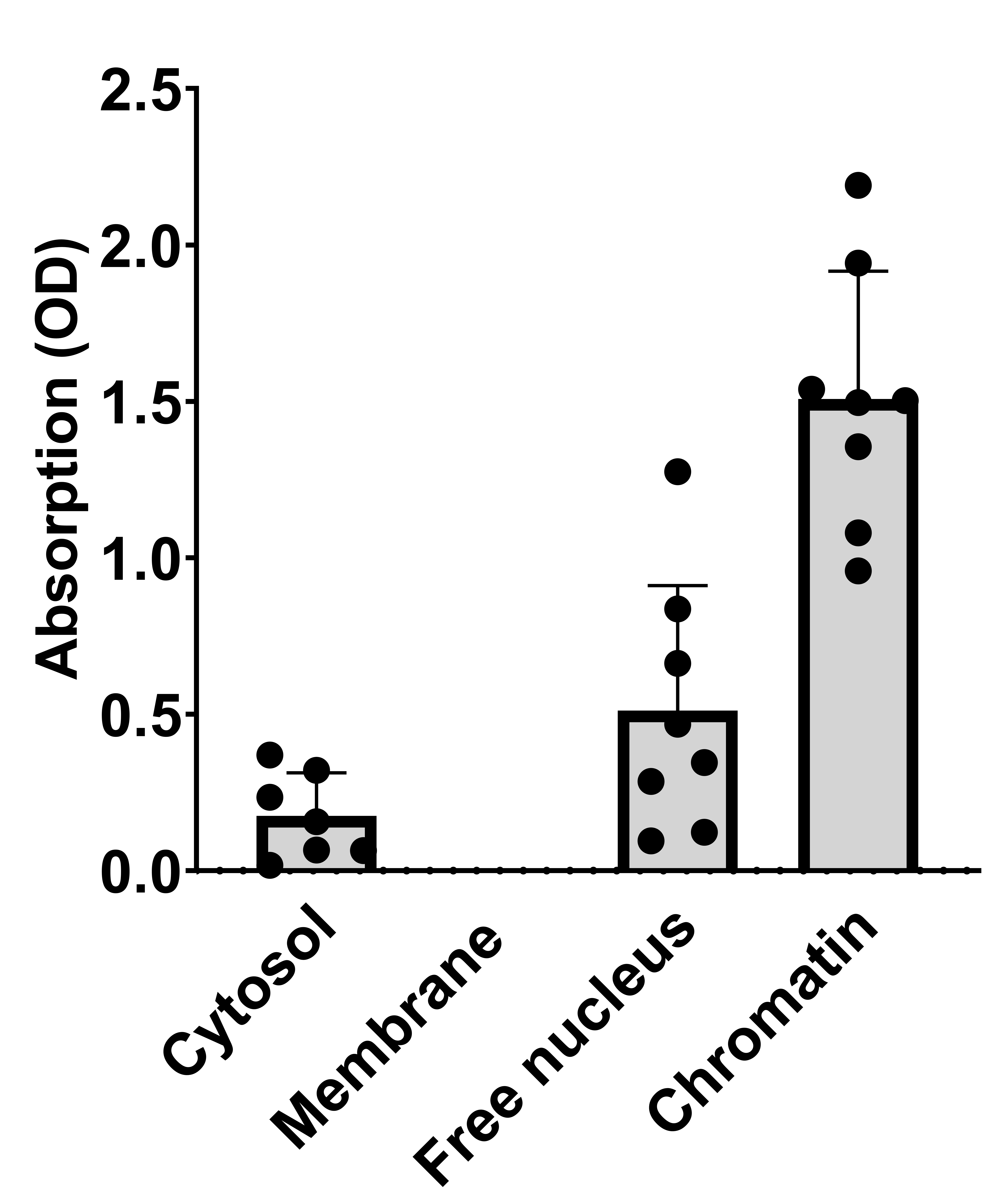

Supplement: Supplementary file 1 [file cancers-15-04096-s001.zip › Figure S8.tif]

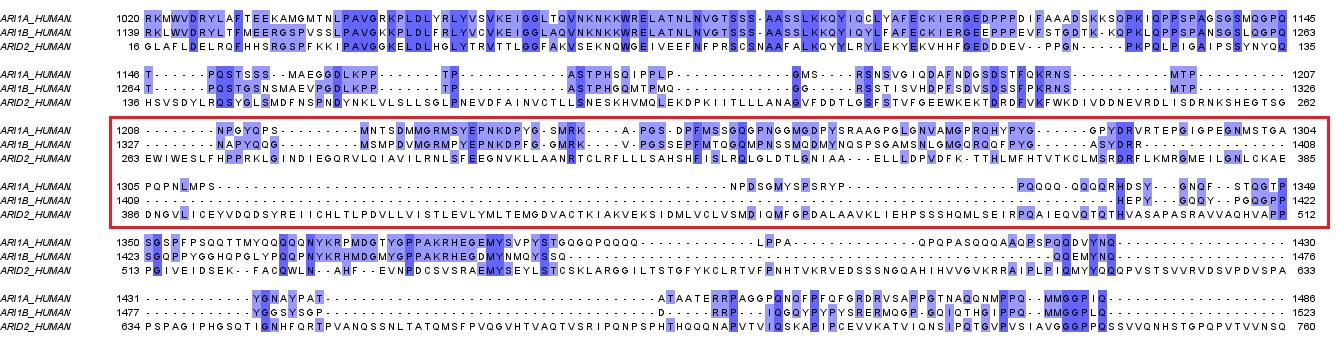

Supplement: Supplementary file 1 [file cancers-15-04096-s001.zip › Figure S9.tif]
